# Supplementary material for: A Non-Sacrificial 3D Printing Process for Fabricating Integrated Micro/Mesoscale Molds
Source: Micromachines (Basel). 2023 Jun 30;14(7):1363. doi: 10.3390/mi14071363 (PMC10385488; doi:10.3390/mi14071363)
Supplement: Supplementary file 1 [file micromachines-14-01363-s001.zip › micromachines-2454569-supplementary.pdf]

Supplementary materials

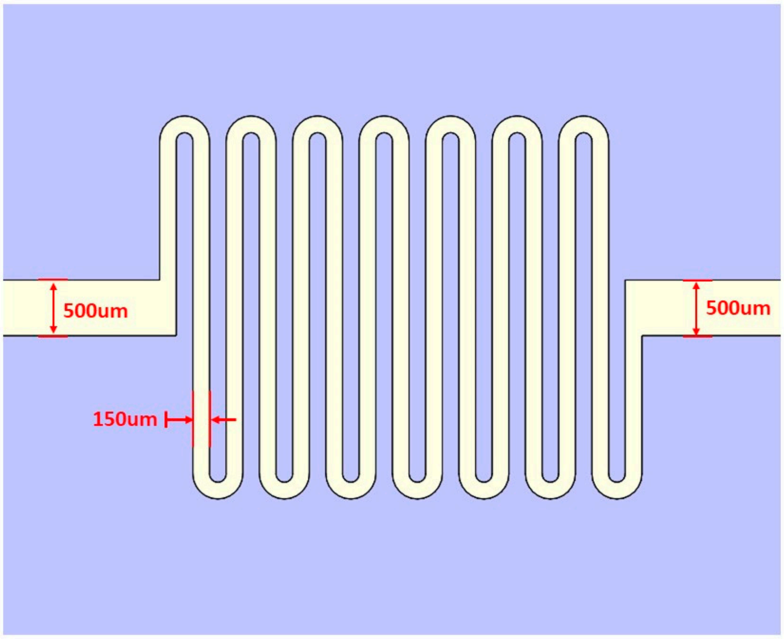

Figure S1. Serpentine channels with two different channel dimensions. Channels rooting from the inlets and connected to the outlet (with 500µm channel width), and mixing channels (150µm channel width).

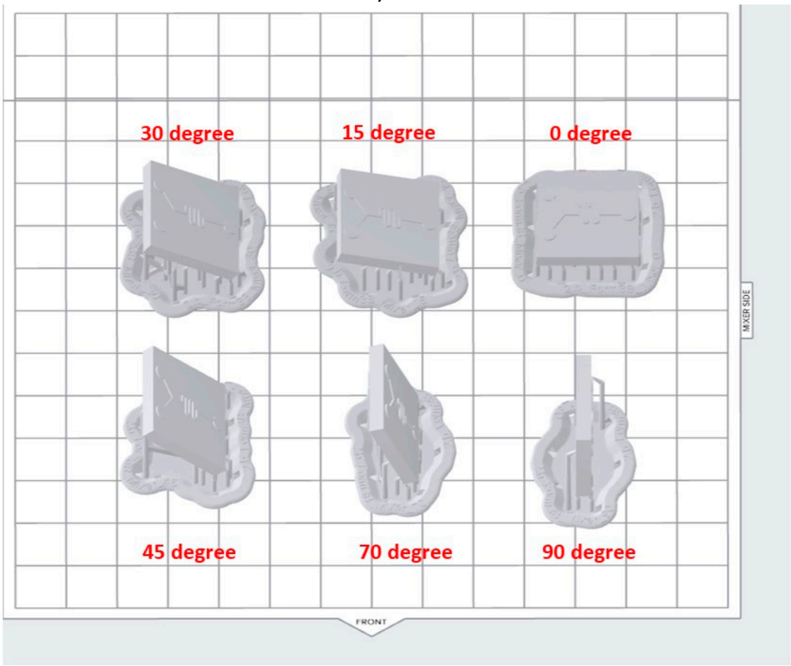

Figure S2. Orientation of the mixer mold on the build platform of MESS 3D printer.

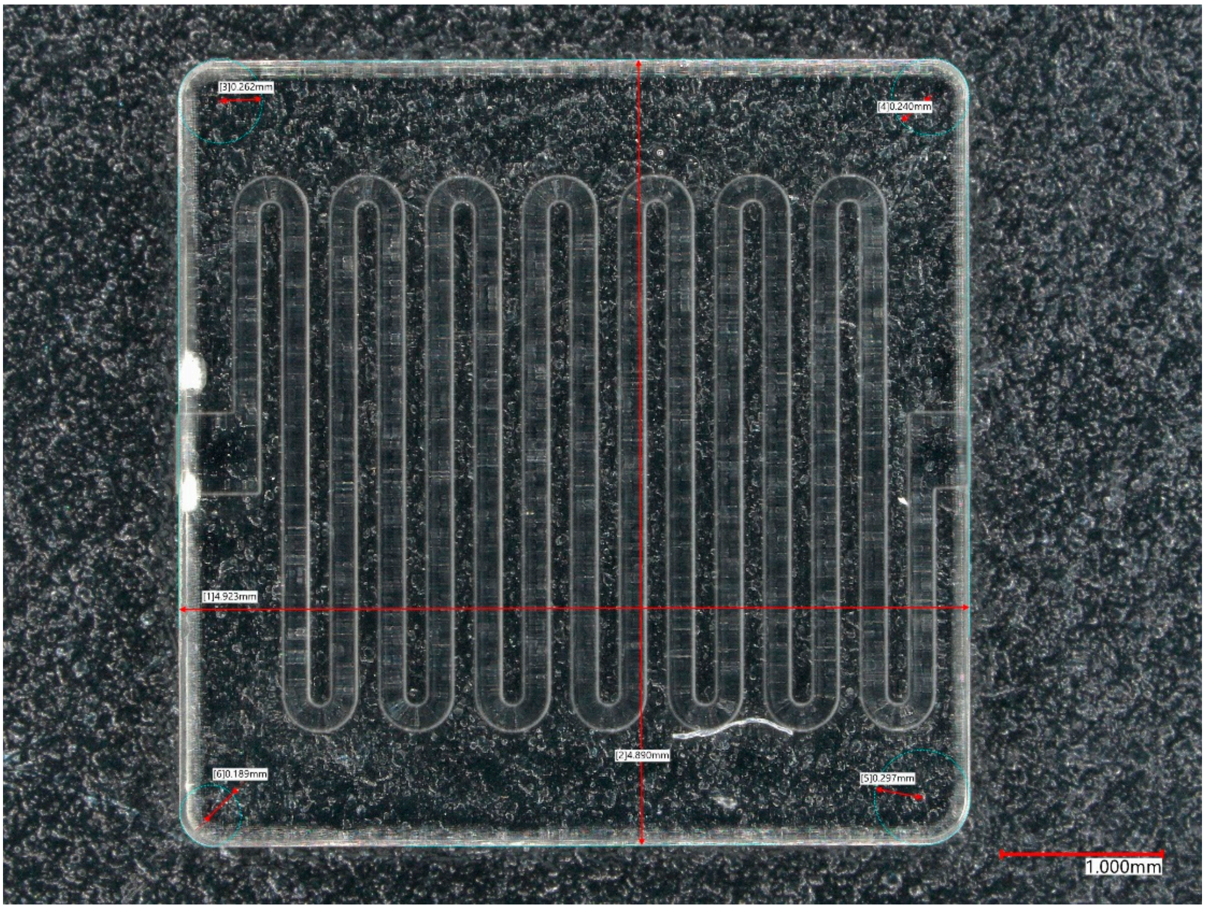

Figure S3. Local characterization of the insert part (serpentine mixer) after microscopic measurements of the base part.

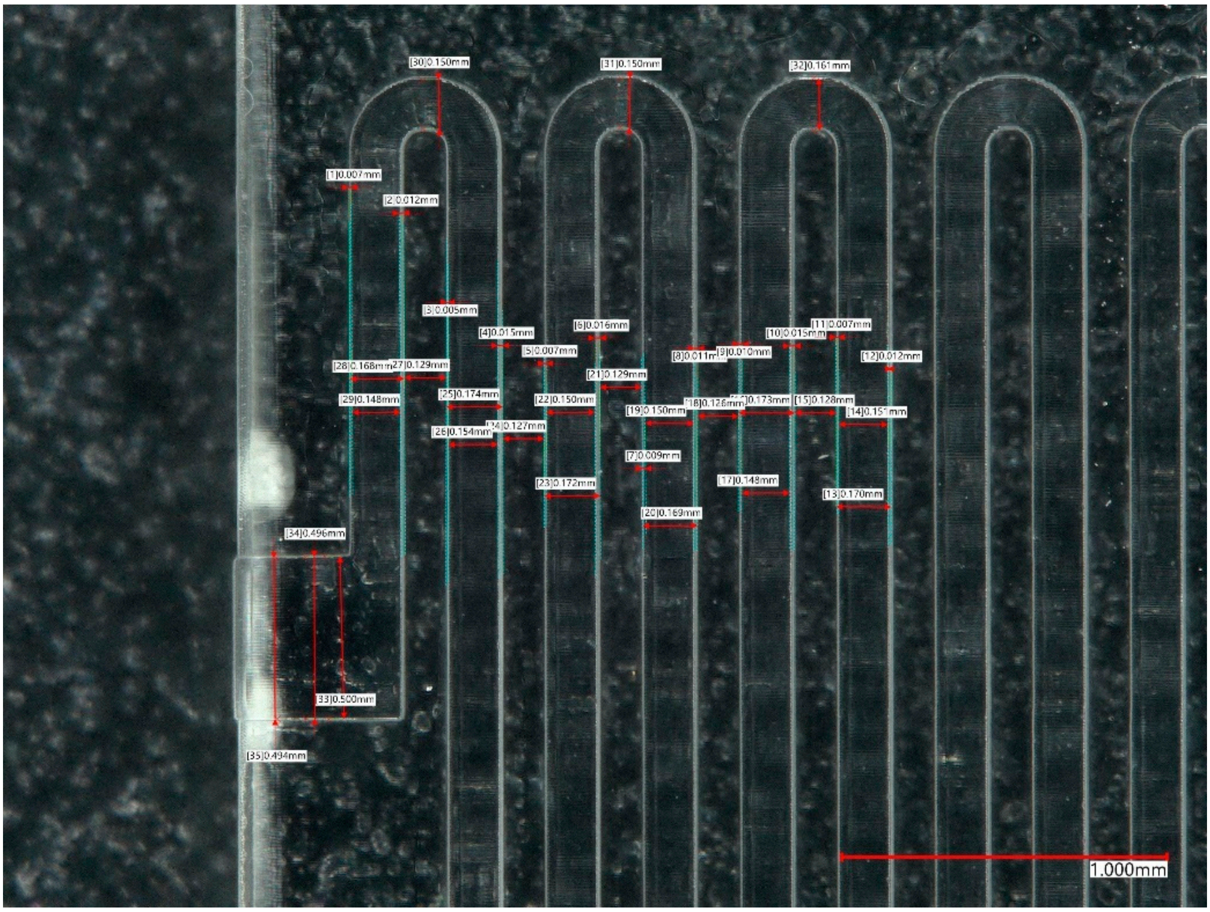

Figure S4. Local characterization of the insert part (serpentine mixer) after microscopic measurements of the base part. The conducted measurements for Figure 10.a and 10.b.
